# Identification of *Acinetobacter radioresistens* in wastewater effluent from a First Nation reserve in Manitoba, Canada

**DOI:** 10.1128/mra.00221-25

**Published:** 2025-07-31

**Authors:** Rudra Patel, Dawn White, Ayush Kumar, Miguel Uyaguari

**Affiliations:** 1 Department of Microbiology, University of Manitoba 468335, Winnipeg, Manitoba, Canada; Indiana University, Bloomington, Bloomington, Indiana, USA

**Keywords:** *Acinetobacter radioresistens*, UV light, effluent, wastewater treatment, First Nation community, whole-genome sequencing

## Abstract

*Acinetobacter radioresistens* was identified in UV-treated wastewater effluent from a First Nation reserve in Manitoba, Canada, by whole-genome sequencing (3,195,655 bp; 41.7% GC) and digital DNA-DNA hybridization with *A. radioresistens* DSM 6976 (average contig nucleotide identity: 90.6%). Draft genome analysis revealed the presence of the clinically relevant carbapenem-resistance gene, *bla*_OXA-23_.

## ANNOUNCEMENT

*Acinetobacter radioresistens* was first isolated from irradiated cotton in 1988 (1) and is the original source of a mobile, clinically relevant, carbapenem-resistance gene *bla*_0XA-23_ (2). Most commonly found as a commensal microbe on healthy human skin (3), it is increasingly being identified in human infections (4). Here, we isolated *A. radioresistens* from wastewater treatment plant effluent at a First Nation reserve located 2 h away from Winnipeg, MB, Canada.

Wastewater (1 L) post-UV treatment was collected in a sterile bottle following standard procedures (5) on 9 September 2022. A 100 µL sample was spread on a Chromocult coliform agar plate and incubated at 37°C for 24 h. A small, white colony was isolated and re-streaked (×3) onto a fresh coliform agar plate and incubated at 37°C for 24 h to ensure colony purity. Antibiotic MIC testing revealed resistance against imipenem (64 µg/mL) following CLSI procedures and breakpoints (6). Genomic DNA was isolated from an overnight culture grown in lysogeny broth using the MasterPure Complete DNA and RNA Purification Kit (LGC BioSearch Technologies). No DNA shearing or size selection was performed. A DNA library was prepared using the Oxford Nanopore Technologies Native Barcoding kit (V14) (SQK-NBD114-24) following the manufacturer’s instructions. The library was sequenced using the MinION with a R10.4.1 flow cell (FLO-MINI114) using the Fast Model base-calling algorithm (400 bps) with a minimum read length of 200 bp without barcode trimming, mid-read filtering, modified base calling, and adapter trimming. All sequence analyses used default parameters unless noted otherwise.

**Fig 1:**
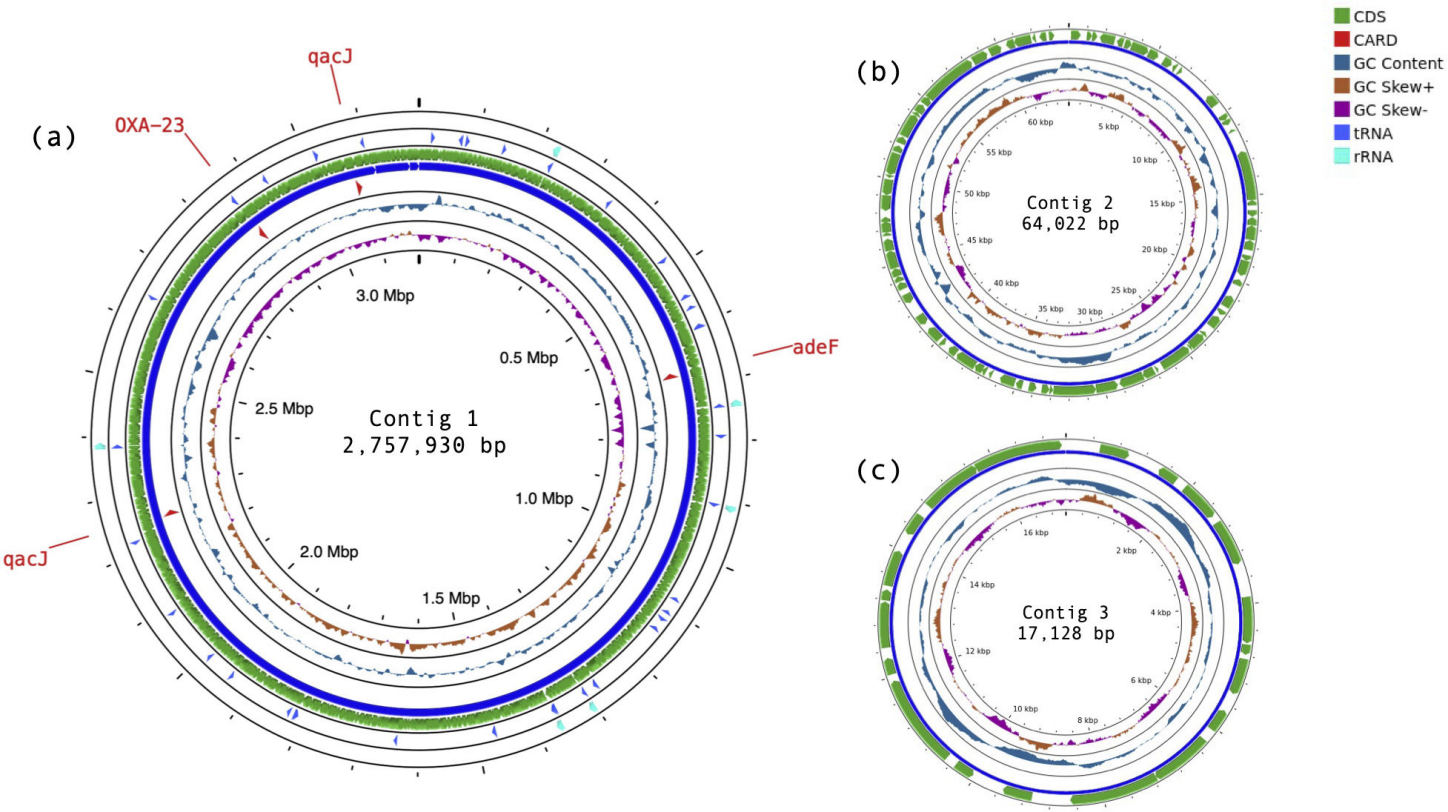
Circular contig maps generated using Proksee (13). (**A**) Contig 1, (**B**) contig 2, and (**C**) contig 3. From the inner ring to the outer ring: GC skew (positive GC skew, mustard; negative GC skew, magenta); GC content (dark blue); Comprehensive Antibiotic Resistance Database (CARD) results (red, annotated); coding DNA sequences (CDS) (green); tRNA (bright blue); and rRNA (turquoise). Analysis of contigs 2 and 3 did not yield tRNA, rRNA, or CARD results.

From 7.65 million raw reads (N_50_: 2.05 kb), 73,803 sequences were assembled using default settings of Flye (v2.9.1-b1780) (7), yielding 47× mean coverage of 3 contigs totaling 3,195,655 bp and GC content of 41.7% (N_50_: 3.11 Mbp): contig 1 comprised 3,114,505 bp, 41.8% GC; contig 2 comprised 64,022 bp, 37.9% GC; and contig 3 comprised 17,128 bp, 37.0% GC (Fig. 1). Alignment of the sequenced genome using rMLST (8) and MiGA (v1.3.21.3) (9) identified our isolate as *A. radioresistens*; alignment using TYGS (v.391) (10) more specifically paired with *A. radioresistens* DSM 6976 and LH6. NCBI MSA alignment of our isolate and LH6 DNA sequences for 16S rRNA (99.5% identity) and *rpoB* (99.7% identity) further strengthened the identity of our isolate. The predicted antibiotic resistance-associated carbapenem-resistance gene, *bla*_OXA-23_ was also present (100% identity). The draft genome was estimated to be 93.4% complete using FastAAI (v0.1.17) (11). Annotation using RASTtk (v1.073) (12) revealed 3,502 CDS in contig 1, 83 CDS in contig 2, and 23 CDS in contig 3, where 3,409 were protein-coding and 93 were RNA genes (18 rRNAs, 75 tRNAs, and 0 ncRNAs).

Resistance-associated genes were found through the Comprehensive Antibiotic Resistance Database (v3.3.0) (14) using RGI *main*; perfect and strict cut-offs revealed *bla*_OXA-23_ and two antibiotic efflux pump genes (Table 1). Antimicrobial-resistant *A. radioresistens* is an emerging burden on the healthcare system (4). Increased surveillance of wastewaters—a documented source of antibiotic-resistant genes (15)—may alert us to potential new AMR bacterial threats.

**TABLE 1:** Antibiotic-resistance related genes of *A. radioresistens* isolate identified using the Comprehensive Antibiotic Resistance Database

Antibiotic resistance related gene	CARD accession	Cut-off	Gene family	Percent length of reference sequence
*bla*OXA-23	ARO:3001418	Perfect	OXA betalactamase	100
*adeF*	ARO:300777	Strict	Resistance nodulation- division efflux pump	99
*qacJ*	ARO:3007014	Strict	Small multidrug resistance efflux pump	102

## Data Availability

The BioSample, BioProject, and SRA numbers for this *A. radioresistens* isolate (SEPT_EFF1) are SAMN42123106, PRJNA1129067, and SRR32145570, respectively.
